# Myocarditis Leading to Severe Dilated Cardiomyopathy in a Patient with Dengue Fever

**DOI:** 10.1155/2015/319312

**Published:** 2015-02-23

**Authors:** Hassan Tahir, Vistasp Daruwalla, Saleem Hayat

**Affiliations:** ^1^Department of Internal Medicine, Conemaugh Memorial Hospital, 1086 Franklin Street, Johnstown, PA 15905, USA; ^2^Department of Internal Medicine, Jinnah Hospital Lahore, Allama Shabbir Usmani Road, Lahore 54700, Pakistan

## Abstract

*Background*. Majority of dengue fever cases follow a benign self-limiting course but recently rare presentations and complications are increasingly seen due to rising burden of disease. Cardiac involvement in dengue fever with fatal outcome is a very rare complication. We report a case of 44-year-old patient who presented with symptoms of severe acute congestive heart secondary to myocarditis induced cardiomyopathy caused by dengue virus infection. *Case Presentation*. A 44-year-old man presented to ER with the complaints of high fever, fatigue, and shortness of breath. Patient was lethargic and blood pressure was low when he was brought to the ER. CXR showed cardiomegaly with pulmonary congestion and echocardiography revealed dilated left ventricle and ejection fraction of 10%. Patient condition worsened and he got admitted to the ICU because of acute hypoxic respiratory failure. Despite aggressive measures, patient died on day 5. *Conclusion*. Dilated cardiomyopathy is a rare complication of dengue myocarditis. Early recognition of acute DCM caused by dengue myocarditis is imperative in the management of dengue fever as early detection and management of cardiac failure can improve the survival of patient.

## 1. Background

Dengue fever is the most rapidly spreading mosquito borne viral illness transmitted by* Aedes* mosquito particularly* Aedes aegypti* [1]. Dengue fever (DF) can cause a wide spectrum of presentations ranging from uncomplicated self-limiting febrile illness to severe dengue including dengue hemorrhagic fever (DHF) and dengue shock syndrome (DSS). Dengue fever is usually caused by 4 distinct serotypes (DEN1, DEN2, DEN3, and DEN4). There has been a dramatic increase in the number of cases of dengue fever recently with almost 50 million people infected every year [2]. With the increasing incidence of disease, an increasing number of cases are being reported with rare complications and atypical presentations. Cardiac complications of dengue fever, though uncommon, have been reported as burden of disease is increasing. A variety of cardiac complications have been recognized, the most common being the myocarditis, though conduction defects and arrhythmias have also been reported [3, 4]. We report a case of dilated cardiomyopathy patient during secondary infection of dengue fever.

## 2. Case Presentation

A 44-year-old male with the past medical history of essential hypertension and tuberculosis presented to the emergency room (ER) with the complaints of high grade fever, fatigue, and shortness of breath. According to patient, he developed high grade fever 1 week ago that was accompanied by myalgias, frontal headache, and generalized weakness. Fever was followed by shortness of breath and exercise intolerance which started 3 days ago and worsened with time. History was positive for mild paroxysmal nocturnal dyspnea and orthopnea though he denied any chest pain, palpitations, cough, or syncope. He also had frequent light headedness and got 1 episode of coffee ground emesis after which he was brought to ER. There was no history of blood in stools or urine. The patient denied any history of diabetes, ischemic heart disease, or hyperlipidemia. The patient was only taking HCTZ for hypertension and did not have any allergies. He was an active smoker with a history of one pack of cigarettes daily but denied use of alcohol or illicit drugs. The family history was positive for myocardial infarction in father at the age of 77. The patient did have a history of recent sick contact with one of his brothers who had dengue fever 3 weeks ago.

Review of patient's past medical records indicated that patient had dengue fever 1 year ago for which he was admitted on the floor but fever got resolved on its own with no complications. At that time patient developed vague chest discomfort for which EKG was done which showed widespread T wave inversions. Cardiac enzymes were mildly elevated and echocardiography showed normal wall thickness and ejection fraction of 58%. Taking into account above findings, a tentative diagnosis of mild dengue myocarditis was made. CXR was normal and patient was discharged. Endomyocardial biopsy and cardiac MRI were not done as the patient had mild myocarditis with normal myocardial functions. Follow-up CXR, Echo, and EKGs were normal and patient remained healthy other than having slight fatigue.

In ER patient was lethargic and seemed anxious due to generalised body aches and headache. On physical examination, vital signs were as follows: blood pressure of 89/52 mmHg, temperature of 103 F, heart rate of 116/min, and respiratory rate of 19. Chest examination revealed reduced breath sounds at lung bases bilaterally with scattered fine crackles in both lungs; no rhonchi or wheeze was heard. Examination of heart was remarkable for normal S1 and S2 with no murmur or clicks; an S3 gallop sound was audible. There was no pallor, scleral icterus, cyanosis, clubbing, or peripheral edema. The rest of the physical examination was unremarkable.

**Figure 1:**
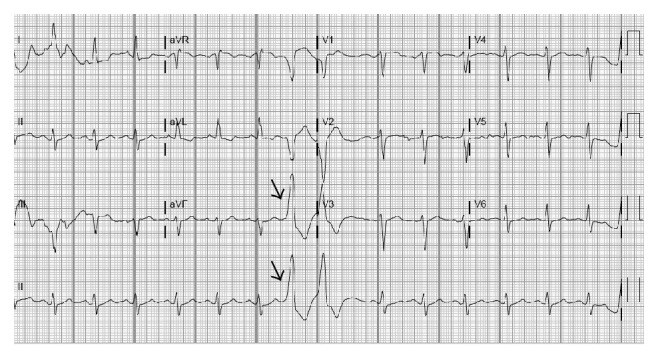
EKG shows normal sinus rhythm with frequent premature ventricular complexes (Arrows).

**Figure 2:**
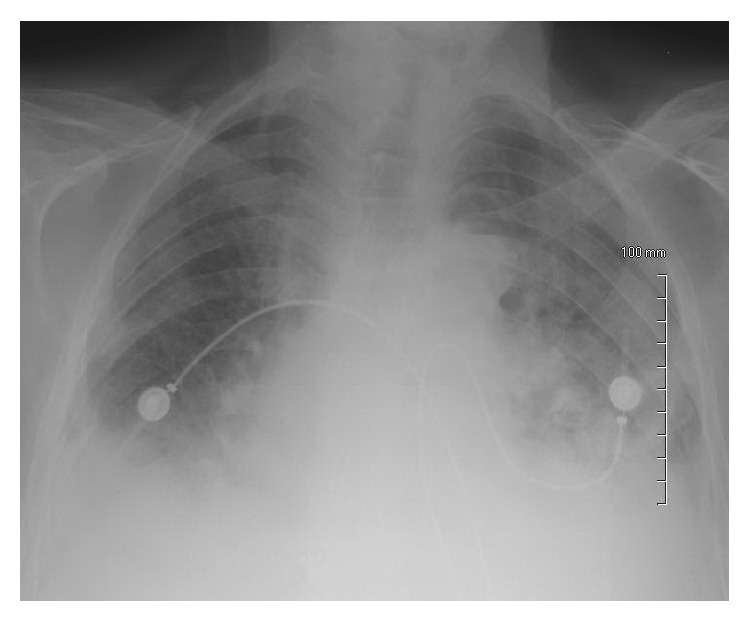
CXR done on 2nd day shows pulmonary edema, cardiomegaly, and bilateral pleural effusions.

**Figure 3:**
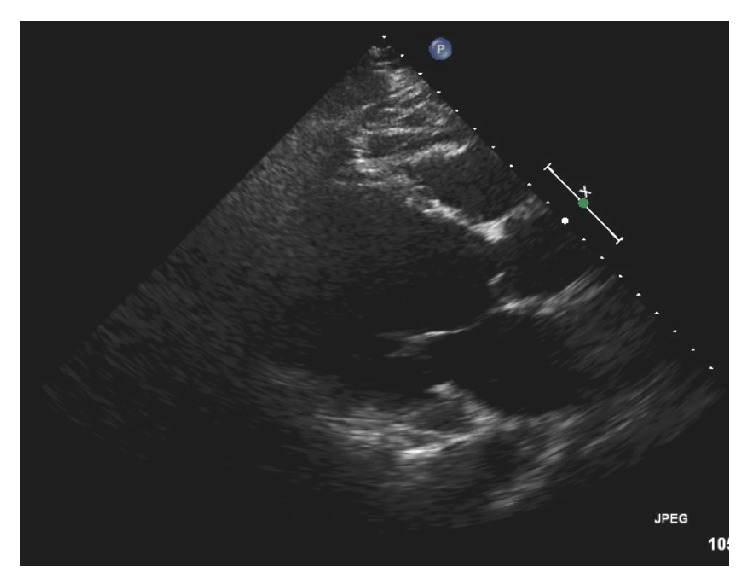
Echocardiogram showing dilated LV and EF of 10%.

**Figure 4:**
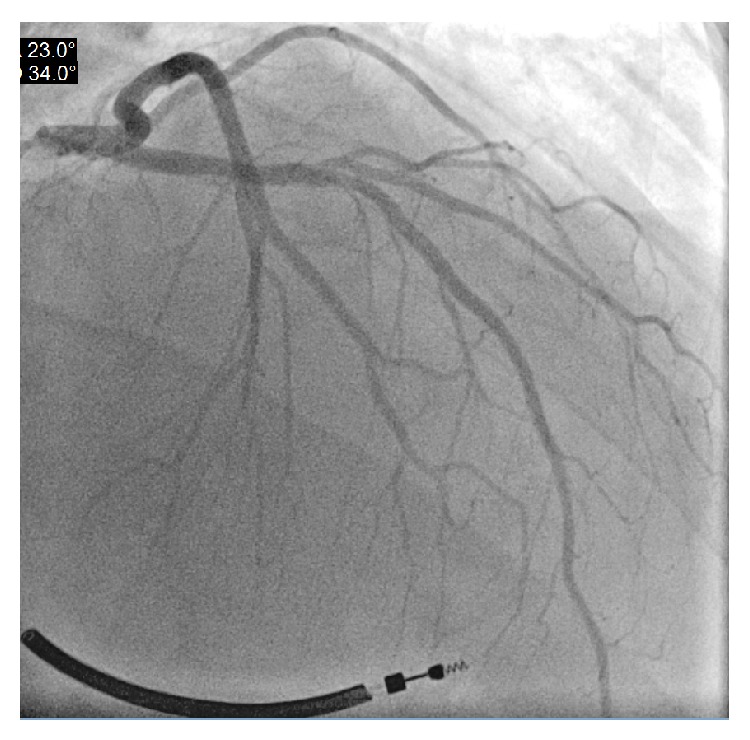
Cardiac catheterization showing normal coronary arteries.

Patient was suspected to have dengue shock syndrome and was immediately admitted on floor and resuscitated with IV fluids. Antiemetics and Tylenol was also given. Routine EKG was done which showed sinus rhythm and frequent premature ventricular contractions (PVC) with no ST or T wave ischemic changes (Figure 1). CXR showed bilateral interstitial edema, cardiomegaly, and pleural effusion in both lungs (Figure 2). The X-ray findings were suggestive of acute congestive heart failure. Elevated BNP (1040) supported the X-ray findings His haematological investigations revealed slight thrombocytopenia (127000), leucopenia (3100), and haemoglobin of 12. Cardiac enzymes were initially slightly elevated but remained stable over the course of hospital stay (Table 1). Urgent transthoracic echocardiography was done which showed ejection fraction of 10% and left ventricle dilation without focal wall motion defect and or focal thinning (Figure 3). Cardiac catheterization revealed EF of 13% and normal coronary arteries (Figure 4). Patient was started on dobutamine infusion along with ACEIs, spironolactone, and Lasix. Blood cultures, ESR, hepatitis panel, thyroid profile, and urine drug screen were all within normal limits. Malarial antigen test and typhoid serology were also negative. Anti-dengue IGM by ELISA was negative at the time of admission but was positive for IGG antibodies at that time. Repeat testing on day 3 showed positive IGM antibodies for dengue. The diagnosis was confirmed with reverse transcriptase polymerised chain reaction (RT-PCR) which was positive for dengue virus serotype 3 (DEN-3). In view of above clinical scenario with positive dengue serology, history of myocarditis during primary dengue infection 1 year ago, and echocardiographic findings, diagnosis of dengue virus induced dilated cardiomyopathy (DCM) was made. Fulminant myocarditis with acute myocardial failure was ruled out as cardiac enzymes were almost normal in the setting of dilated cardiac chambers on Echo.

Patient condition worsened gradually and he developed acute hypoxic respiratory failure for which he was intubated on day 4. Patent developed severe thrombocytopenia and despite platelet transfusions he developed massive lower GI bleed leading to refractory shock. Patient was transfused 2 units of PRBC and platelets, but despite aggressive resuscitation and intensive care, patient died on day 5.

## 3. Discussion

DCM is a disease of heart muscle characterized by muscle weakening leading to contractile dysfunction and not associated with other major causes of cardiac disease such as ischemic heart disease, hypertension, and valvular disease [5, 6]. LV dilation and systolic dysfunction (<55%) with normal left ventricular wall thickness are essential for diagnosis [7]. DCM is the most common reason of heart transplantation and 3rd most common cause of congestive heart failure with about 400,000 to 550,000 cases annually [8]. Presenting complaints may vary from asymptomatic and fatigue to severe shortness of breath and even life threatening arrhythmia resulting in death. Exertional dyspnea and exercise intolerance are the most common presentations though some patients are identified incidentally with asymptomatic cardiomegaly on CXR [9]. Nevertheless, symptoms are good indicator of severity of DCM. DCM has many causes which include toxins, metabolic, infections, and inflammation but majority of cases have no cause and cardiomyopathy is deemed idiopathic.

Viral infection is the most common cause of acute myocarditis which can lead to DCM [9]. Many viruses can affect the myocardium but the most common virus affecting myocardium in adults is adenovirus. Cardiac complications in dengue fever, previously thought very rare, are now increasingly observed. Myocarditis is the most common cardiac manifestation in dengue fever. A cohort study in Sri Lanka showed increasingly asymptomatic myocardial involvement with no long term complications [10].

Dengue fever is a mosquito borne infection spread by* Aedes aegypti* mosquito. Primary infection with dengue virus provides lifelong immunity against that serotype. Subsequent infection with a different serotype can result in a more severe and rarely life threatening disease that includes dengue haemorrhagic fever and dengue shock syndrome. It is speculated that antibody dependent enhancement (ADE) is the mechanism responsible for severe disease in secondary infection [11, 12]. Infection with dengue virus is asymptomatic in majority of cases but it may cause a spectrum of diseases ranging from self-limiting febrile illness to serious hemorrhagic manifestations and refractory shock. Dengue shock syndrome can present with refractory hypotension, large pleural effusion, ascites, and even pulmonary edema. Although most symptomatic dengue infections follow an uncomplicated course, complications and unusual manifestations are increasingly being reported due to rising burden and increased awareness. Dengue fever can rarely affect the heart and present with variety of presentations. Arrhythmias, conduction blocks, myocarditis, and even acute myocardial failure has been reported [4, 13, 14]. The main mechanism of dengue myocarditis is still unknown though both direct viral infection and immune mediated damage have been suggested to be the cause of myocardial damage [15]. The low incidence of dengue myocarditis is because of the reason that patients are most of the times asymptomatic or have vague symptoms and diagnosis is easily missed. Studies showed that asymptomatic myocardial involvement in dengue fever is much more common than previously thought but almost all cases of dengue myocarditis are self-limiting and severe myocarditis leading to DCM is extremely rare.

Our patient had possible myocarditis during primary infection. Diagnosis was not confirmed with endocardial biopsy and cardiac MRI due to vague symptoms and normal cardiac functions. Mild chronic myocarditis may have led to dilated cardiomyopathy over the course of time. The patient got secondary infection with dengue fever and this time he presented with acute congestive heart failure secondary to dengue viral induced DCM. Cardiac catheterization showed normal coronary arteries so ischemic dilated cardiomyopathy was ruled out. Patient did not have a history of congenital heart disease and his EKG and CXR were normal 1 year ago so other nonischemic causes of cardiomyopathy were less likely. Patient tested positive for dengue virus and diagnosis was confirmed with PCR. Patient's above clinical picture in the presence of dengue infection led to the diagnosis of dengue induced DCM. Treatment of dengue DCM is the same as congestive heart failure; there is no antiviral therapy for dengue virus and treatment is largely supportive. Our patient failed to respond to conventional heart failure treatment and unfortunately he developed severe dengue haemorrhagic fever leading to refractory shock. Despite aggressive measures, his condition worsened leading to his death within a week. DCM with acute congestive heart failure and DSS/DHF together has a worse prognosis and has a higher mortality rate. Dengue induced DCM is easily missed as acute DCM can mimic dengue shock syndrome and patient presenting with DCM can be very young.

## 4. Conclusion

Dengue fever can have varied and rare presentations. Dilated cardiomyopathy though very rare but can occur in dengue fever. Asymptomatic chronic myocarditis may be the possible underlying mechanism leading to DCM. Possibility of dengue virus induced dilated cardiomyopathy should always be considered if a dengue fever patient has refractory shock and signs and symptoms of congestive heart failure. Early detection, prompt resuscitation in ICU, and avoidance of aggressive IV fluids therapy are crucial life saving measures and can improve the survival of patient. Treatment of DCM caused by dengue virus myocarditis is the same as standard heart failure therapy with no role of antivirals, immunosuppressants, and steroids.

## Figures and Tables

**Table 1:** Investigations.

Labs	Day 1	Day 2	Day 3	Day 4	Day 5
Hb (g/dL)	12	11.5	11.7	10.1	10.4
TLC/cumm	3000	2700	2855	2933	2912
Platelet/cumm	127000	55000	22000	8000	13000
CKMB (ng/mL)	57	60		58	
Troponin I (ng/mL)	0.06	0.05		0.04	
BNP (pg/mL)	1040		1217		1425
BUN (mg/dL)	27	33	39	44	57
Creatinine (mg/dL)	1.1	1.0	1.1	1.5	1.8
Dengue IgM	−ive		+ive		
Dengue IgG	+ive		+ive		

## Abbreviations

DF: Dengue fever
DHF: Dengue hemorrhagic fever
DSS: Dengue shock syndrome
DCM: Dilated cardiomyopathy
RT-PCR: Reverse transcriptase polymerised chain reaction
ER: Emergency room
PRBC: Packed red blood cells.
